# Morphometric changes after superomedial pedicle wise pattern reduction mammoplasty – what we plan and where we end up

**DOI:** 10.1016/j.jpra.2026.08.031

**Published:** 2026-08-25

**Authors:** Thomas Holzbach, Silke Graul, Aljosa Macek, Alvija Kucinskaite, Alexander Kobler, Sebastian Leitsch, Rafael Loucas, Michael-Alexander Pais

**Affiliations:** a Department of Hand and Plastic Surgery, Thurgau Hospital Group, Frauenfeld, Switzerland; b Division of Hand, Plastic and Aesthetic Surgery University Hospital, LMU Munich, Germany

**Keywords:** Reduction mammoplasty, Breast reduction, Breast symmetry, Anthropometric measurements, Lower pole expansion, Bottoming out

## Abstract

Superomedial pedicle–based reduction mammoplasty using a Wise-pattern skin resection is a well-established technique for breast reduction. However, long-term data on morphometric changes – such as nipple position shifts and lower pole expansion – remain limited. This study evaluated postoperative morphometric changes and their predictors at 3, 6, and 12 months after surgery, compared with intraoperative planning measurements.

A retrospective cohort study based on prospectively collected routine clinical measurements was conducted in 100 female patients (≥17 years) who underwent primary bilateral reduction mammoplasty using a superomedial pedicle and Wise pattern. Pre- and postoperative data included demographics, comorbidities, resection weights, 3D volumetric assessments, and anthropometric measurements: midclavicle-to-nipple distance (MCN), lower areolar border–to–inframammary fold distance (IMFA), and areolar diameter (DA). Complications were graded according to the Clavien-Dindo classification. Generalized linear and logistic regression models were used to assess associations between patient- and surgery-related variables and postoperative outcomes.

The median age was 39.5 years. Mean resection weights were 430 g (right) and 450 g (left), with 54% of cases ranging between 200 and 499 g. Median postoperative breast volumes were 630 cc at 3 months, 536 cc at 6 months, and 605 cc at 12 months. Minor complications (grade I) occurred in 15% of patients, and one grade IIIb event required reoperation. At 12 months, minimal nipple descent was observed (mean MCN increase of 1.01 cm), along with mild lower pole expansion (mean IMFA increase of 1.27 cm) and a slight increase in areolar diameter (0.25 cm). Higher postoperative breast volume was associated with greater morphometric changes, whereas larger resection weights (500–2000 g) and higher BMI predicted increased lower pole expansion.

Postoperative morphometric changes were significantly associated with breast volume, resection weight, age, and BMI, underscoring the importance of individualized surgical planning and long-term follow-up to optimize aesthetic outcomes.

## Introduction

Postoperative contour change after reduction mammaplasty is multifactorial and progressive.^1^, ^2^, ^3^ In superomedial-pedicle Wise-pattern reductions (SMP-Wise), breast shape may evolve through a combination of soft-tissue remodeling, skin-envelope relaxation, and parenchymal redistribution manifesting clinically as inferior-pole elongation (“bottoming-out”), subtle caudal migration of the nipple-areola-complex (NAC), and changes in areolar diameter. Although these phenomena are well recognized, their natural history and determinants are not fully elucidated.^4^, ^5^, ^6^, ^7^ Critically, even low-grade, static descent can perpetuate further inferior-pole strain and accentuate pseudoptosis, thereby altering the long-term aesthetic endpoint and, in selected cases, precipitating revision.^2^^,^^3^

To describe and monitor these changes, authors have adopted reproducible morphometric descriptors rather than purely qualitative impressions.^8^ Linear anthropometry – particularly the distance from the lower areolar border to the inframammary fold (IMF) as a surrogate of inferior-pole length, and NAC height measured from fixed skeletal or soft-tissue landmarks, offers a standardized vocabulary for longitudinal follow-up and interstudy comparison.^9^

Such measures capture the dimensions most closely linked to perceived “youthfulness” of contour and symmetry, domains to which NAC position contributes disproportionately. Refinement of intraoperative standardization has paralleled this shift toward measurement, with simple but effective tools (e.g., laser-level projection) reducing vertical NAC asymmetry and translating planned targets more faithfully into realized outcomes.^10^

Concurrently, three-dimensional (3D) surface imaging has matured from a research modality into a practical instrument for routine outcomes assessment. Multiple validation studies demonstrate strong agreement between 3D volumetry and reference standards, alongside excellent repeatability for volume and symmetry, endorsing its role in preoperative planning and postoperative surveillance.^11^, ^12^, ^13^, ^14^, ^15^, ^16^

Importantly, methodological work has emphasized that patient position significantly influences volumetric readouts: supine acquisition systematically underestimates upright (standing or seated) volume. Protocolized, posture-consistent imaging - both intraoperatively and at follow-up - is therefore essential to avoid spurious longitudinal inferences and to enable robust comparison across time points.^17^^,^^18^

These technical foundations have catalyzed a transition from anecdote to quantification in the study of breast shape evolution. Despite the ubiquity of SMP-Wise and the growing availability of objective tools, key knowledge gaps remain.^17^, ^18^, ^19^ Prior series often pool techniques, lack predefined follow-up windows, or do not align anthropometry with volumetry, thereby limiting insights into when planned intraoperative geometry tends to diverge from observed morphology, and which early signals meaningfully forecast later breast shape. Emerging experience suggests that an early postoperative checkpoint around the time when edema resolution and soft-tissue accommodation stabilize (approximately three months) provides pragmatic prognostic value for subsequent contour behavior.^17^, ^18^, ^19^

Comparative data further imply that pedicle design and skin-envelope tailoring may modulate long-term morphometrics; however, heterogeneity in definitions, imaging posture, and timing has constrained direct comparisons and underscores the need for stan/rdized, measurement-driven protocols.^20^^,^^21^

This study provides a measurement-based evaluation of postoperative breast morphology after superomedial pedicle Wise-pattern reduction. Standardized anthropometry (inferior-pole length, NAC height, areolar diameter) was combined with posture-controlled 3D imaging to assess temporal morphometric changes and examine associations between patient characteristics, resection weight, postoperative volume, and shape evolution using multivariable models. Particular emphasis was placed on correlations between early postoperative volume (3 months) and later morphometric changes. Using standardized metrics and imaging protocols, this study aims to inform structured morphometric follow-up and surgical planning.

We hypothesized that early postoperative breast volume measured three months after surgery would be associated with subsequent morphometric changes, particularly inferior pole elongation, following superomedial pedicle Wise-pattern reduction mammoplasty.

## Methods

This retrospective single-center cohort study included 100 consecutive female patients (≥17 years) who underwent primary superomedial pedicle Wise-pattern reduction mammoplasty at Thurgau Hospital Group (Switzerland) between July 2023 and December 2024. Patients with complete pre- and postoperative data were included; those with incomplete records or prior breast surgery were excluded.

All preoperative, intraoperative, and postoperative anthropometric measurements were routinely obtained as part of our institutional clinical protocol for patients undergoing reduction mammoplasty and were not collected specifically for this study. The present investigation therefore represents a retrospective analysis of prospectively documented routine clinical data. Postoperative breast volume was assessed using the Vectra H2 three-dimensional photogrammetric imaging system with patients in the upright standing position following a standardized imaging protocol.^10^^,^^16^, ^17^, ^18^

Baseline demographics, surgical details, and anthropometric measurements were recorded. Anthropometric parameters included the midclavicular notch-to-nipple distance (MCN), defined as the linear distance from the medial clavicular notch to the nipple; the inferior areolar border-to-inframammary fold distance (IMFA), defined as the vertical distance from the inferior border of the areola to the inframammary fold; and the areolar diameter (DA), defined as the maximal horizontal diameter of the nipple–areola complex.

Preoperative and intraoperative anthropometric measurements were obtained directly by trained members of the surgical team using a sterile measuring tape and sterile ruler according to a standardized institutional protocol.^10^^,^^16^, ^17^, ^18^ Following tissue resection, breast reshaping, and temporary skin closure, intraoperative measurements were performed with the patient positioned in a semi-upright sitting position to simulate the postoperative standing breast contour. These measurements represented the intended postoperative breast morphology and served as the reference values for subsequent postoperative comparisons.

Postoperative changes at 3, 6, and 12 months were calculated relative to the intraoperative measurements. Associations were analyzed using generalized linear models and logistic regression. Statistical analyses were performed using Jamovi (version 2.3).

### Baseline demographic data

The collected patient-related data included age, obesity (defined as a body mass index (BMI) ≥ 30 kg/m²), a history of regular nicotine use, diabetes mellitus, arterial hypertension, peripheral artery disease, and other pre-existing conditions such as adolescent idiopathic scoliosis (AIS) and prior spine surgeries.

Ethnicity was not routinely documented within our institutional database and was therefore unavailable for retrospective analysis.

### **Treatment-**specific **characteristics of patients undergoing reduction mammoplasty**

Regarding treatment-specific characteristics, the study included 100 female patients who underwent primary bilateral reduction mammoplasty. All procedures were performed by experienced consultant plastic surgeons using a standardized operative technique.

The operation was performed using a superomedial glandular pedicle combined with a Wise-pattern skin excision. Parenchymal resection was predominantly carried out from the inferior and inferolateral breast tissue while preserving the superomedial pedicle to maintain vascularity of the nipple–areola complex and upper pole fullness. Skin closure was performed using a standard inverted-T closure technique.

Resected tissue weight per breast was evaluated, and the average resection weight was calculated. These weights were categorized into smaller resections (100–499 g) and larger resections (500–2000 g) based on classifications from existing literature.^22^^,^^23^ Breast volume was assessed in cubic centimetres (cc) using three-dimensional photogrammetric analysis at 3, 6, and 12 months postoperatively using the Vectra H2 volumetric imaging device. Measurements were obtained in an upright standing position following a standardized imaging protocol.^16^ The 3-month postoperative assessment was predefined as the primary volumetric endpoint, reflecting measurement accuracy after resolution of postoperative edema and prior to late postoperative changes. Volumetric measurements at 6 and 12 months were used to assess temporal stability of breast volume. Associations between postoperative morphometric changes over time, tissue resection, and volumetric symmetry were analyzed using validated volumetric assessment methodologies.^16^

Postoperative complications were categorized using the Clavien-Dindo classification^24^ into three grades: grade I encompassed minor wound healing issues that required no additional interventions, including wound healing disorders managed with extended dressing changes or local wound debridement during routine follow-up visits. Grades II, IIIa, and IIIb included complications necessitating surgical interventions, such as hematoma evacuation or secondary wound closure, excluding scar revisions.

### **Study** outcomes

The primary outcome was postoperative inferior pole elongation, defined as the change in the distance between the lower areolar border and the inframammary fold (IMFA) at 12 months relative to intraoperative measurements.

Secondary outcomes included changes in midclavicular notch-to-nipple distance (MCN), areolar diameter (DA), postoperative breast volume at 3, 6, and 12 months, and the association of these measurements with patient-related and procedure-related variables.

### Statistical **analyses**

Frequency distribution tables were used to provide descriptive statistics for the data. The primary aim was to analyze correlations and associations between patient characteristics and predefined outcomes, including median breast volume at 3, 6, and 12 months post-surgery and average resection weight variations ranging from 500 to 2000 g. Due to the skewed data distribution, a generalized linear model (GLM) with a negative binomial distribution and log link was applied to address significant overdispersion. Incident rate ratios (IRRs) were calculated for relevant patient characteristics (age, BMI ≥ 30 kg/m², nicotine use, and other risk factors such as AIS and previous spine surgeries), grade I complications, and anthropometric measurements (average of MCN, lower areola border to IMF, and DA distance at 12 months).

Count variables were expressed as mean ± standard deviation (SD), age as median ± interquartile range (IQR), and categorical variables as counts and percentages. Statistical significance was defined as p < 0.05.

Only patients with complete datasets at predefined follow-up intervals were included in the final analysis. No imputation for missing data was performed.

All statistical analyses were conducted using Jamovi software (version 2.3, Sydney, Australia).

### Measures **to address bias**

To minimize selection bias, consecutive patients meeting eligibility criteria during the study period were included. Measurement bias was reduced through standardized anthropometric documentation and posture-controlled volumetric acquisition using a predefined imaging protocol. Nevertheless, as a retrospective single-center study, residual confounding and unmeasured variables cannot be excluded.

### Reporting **standards**

The study was conducted in accordance with the STROBE guidelines for cohort studies. The completed checklist is provided as supplementary material.

## Results

### Baseline characteristics and demographic data

A total of 100 consecutive eligible female patients with complete preoperative and postoperative follow-up data were included in the final analysis. The median age was 39.5 years (IQR 17–80). The most common comorbidities at admission were obesity (18%), nicotine use (3%), arterial hypertension (6%), and other conditions (10%), including adolescent idiopathic scoliosis (AIS) and prior spinal surgery. A detailed overview of baseline characteristics and comorbidities is presented in Table 1.

**Table 1:** Baseline demographic data.

Baseline demographic data (n = 100)	
Patient characteristics	N^1^ [counts] (Percentage [%]), or median (IQR^1^)
Age	39.5(17 and 80)
BMI* ≥ 30kg/m^2^**	18^18^
Nicotine consumption	3^3^
Diabetes mellitus	0 (0)
Arterial hypertension	6^6^
Peripheral artery disease	0 (0)
Others (e.g., AIS**, spine surgeries)	5^10^

Continuous variables ± IQR, binary variables respectively, frequency tables. * Body Mass Index (BMI), ** Adolescent idiopathic scoliosis (AIS). **^1^** N = total number of patients, IQR = Interquartile range.

### **Treatment-**specific **characteristics of patients undergoing reduction mammoplasty**

A cohort of 100 patients underwent primary reduction mammoplasty using the Wise pattern resection technique. The median resection weight was 430 g (SD: 315) on the right side and 450 g (SD: 325) on the left. The average resection weight was categorized into two ranges: 200–499 g and 500–2000 g, with means of 54 (SD: 54) and 46 (SD: 46), respectively (Table 2). Median breast volumes measured at 3, 6, and 12 months postoperatively were 630 (SD: 155), 536 (SD: 141), and 605 (SD: 165), respectively (Table 2).

**Table 2:** Therapy characteristics.

Therapy specific characteristics (n = 100)	
Patient characteristics	N^1^ [counts] (Percentage [%]), or median (SD^1^)
Resection weight [g] Right Left	430 (315) 450 (325)
Average resection weight [counts] ranging from 200 to 499 ranging from 500 to 2000	54 (54) 46 (46)
Median breast volume [cc] at 3 months	630 (155)
Complications (Clavien-Dindo classification) Grade I Grade II and IIIa Grade IIIb	15^15^ 0 (0) 1^1^

Continuous variables ± SD, binary variables respectively, frequency Tables 1. **^1^** N = total number of patients, SD = standard deviation.

Further evaluation of complications revealed that 15% of patients experienced grade I complications, which included minor wound healing issues that required no additional intervention or wound healing disorders managed with prolonged wound dressing or local wound debridement during routine postoperative follow-ups. Moreover, one patient (1%) experienced grade IIIb complications, requiring surgical intervention (Table 2).

### Variations in morphometric measurements observed at 3, 6 and 12 months postoperative follow-up

Anthropometric variations were assessed at 3, 6, and 12 months postoperatively using a standardized anthropometric measurement data sheet. These measurements included the distances of the medial clavicular notch (MCN), the lower areola border to the inframammary fold (IMF), and the areolar diameter for each breast, as well as their averages. Table 3.

**Table 3:** Morphometric variances postoperatively, compared to intraoperative anthropometric values.

Morphometric variances postoperatively, compared to intraoperative anthropometric values (n = 100)				
Anthropometric characteristics [cm] – Patient-level median for both sides	Intra-OP	3 Mo	6 Mo	12 Mo
	Mean (SD^1^)			
MCN*	21.5 (2.21)	0.90 (2.50)	1.01 (2.36)	1.01 (2.40)
Lower areola border to IMF**	6.00 (0.95)	0.97 (1.44)	1.55 (1.44)	1.72 (1.54)
DA***	3.80 (0.16)	0.23 (0.45)	0.33 (0.67)	0.25 (0.48)

Continuous variables ± SD, frequency tables. Missing data, for the average of MCN: 3 to 4, lower end of areola to IMF: 4 to 5, areolar diameter: 4 to 5. MCN* = midclavicular distance, IMF** = inframammary fold, DA*** = areolar diameter. **^1^** SD = standard deviation.

**Fig. 1:**
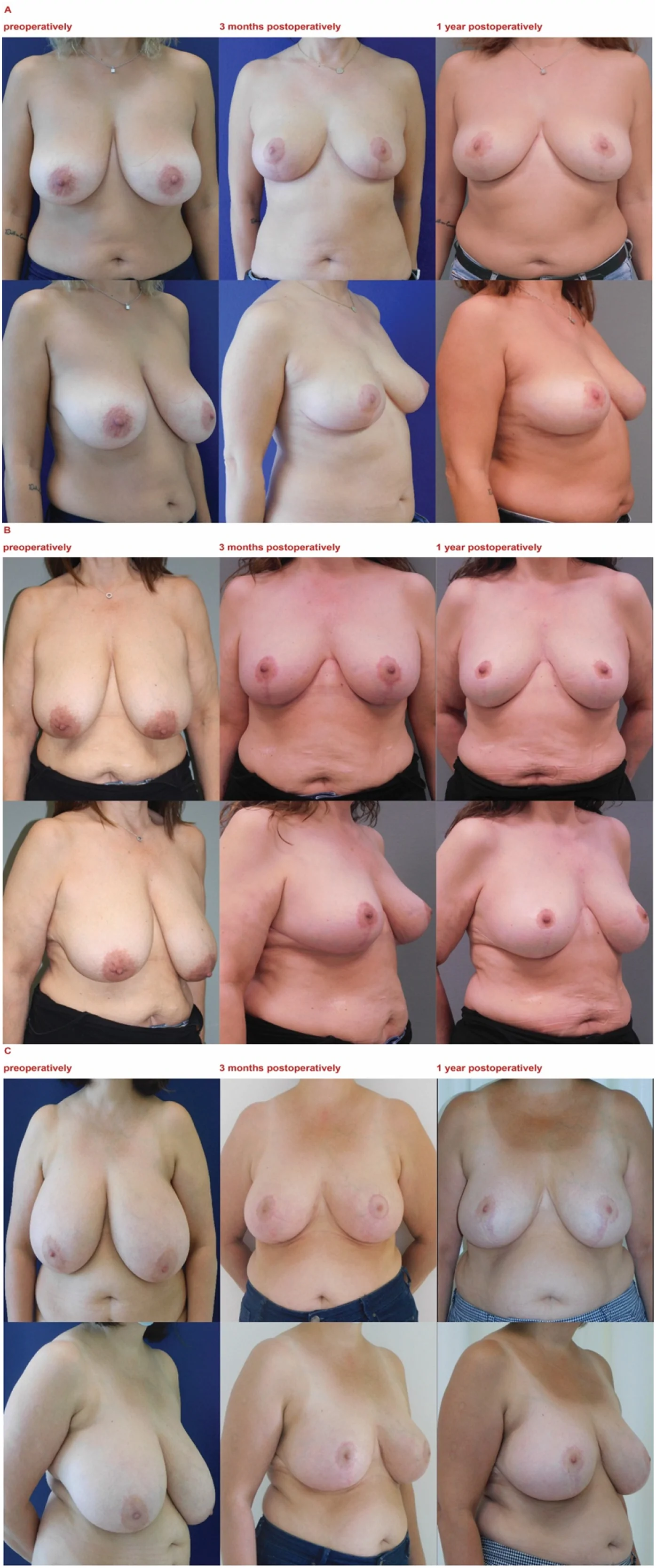
Representative clinical photographs demonstrating longitudinal morphometric changes following reduction mammoplasty using a superomedial pedicle and Wise-pattern skin resection. Panels A–C show three representative patients photographed preoperatively, at 3 months, and at 1 year postoperatively (frontal and oblique views) following reduction mammoplasty using a superomedial pedicle with Wise-pattern skin resection. Standardized anthropometric measurements were obtained at 3 and 12 months, including the distance from the medial clavicular notch to the nipple–areola complex (MCN) and areolar diameter (DA), within a retrospective cohort of 100 patients aged ≥17 years. Patient-specific trends are illustrated: Patient A showed MCN increases of 1.05 and 0.9 cm, with DA measurements of 0.7 and 0.4 cm at 3 and 12 months, respectively; Patient B showed MCN increases of 1.4 and 1.0 cm, with DA values of 0.2 and 0.4 cm; Patient C demonstrated MCN increases of 1.1 cm and DA values of 0.2 cm at 6 and 12 months. Overall, these findings demonstrate stable long-term breast shape with only mild inferior pole descent (“bottoming-out”) and partial regression of areolar diameter at one year postoperatively.

Over the 12-month period, the MCN distance increased by an average of 1.01 cm (SD: 2.40), reaching a maximum increase of approximately 1 cm. The distance from the lower areolar border to the inframammary fold also demonstrated a mean increase of 1.72 cm (SD: 1.54) (Fig. 1).

In contrast, the areolar diameter initially increased by 0.33 cm (SD: 0.67) at 6 months but subsequently decreased by 0.25 cm (SD: 0.48) at 12 months, resulting in a mean areolar diameter of 3.98 cm (SD: 0.45) at 12 months (Fig. 1).

### Median breast volume at 3 months was positively associated with changes of the lower breast pole at 12 months post surgically

We first investigated potential correlations between differences of the median breast volume at 3 months post surgically and various predictive factors. These factors were age, BMI ≥ 30 kg/m2, nicotine consumption, and grade I complications. Correlations were also analyzed for morphometric changes postoperatively, such as average of MCN, lower areola border to IMF, and DA distance at 12 months (Table 4).

**Table 4:** Median breast volume at 3 months post surgically, with different associations.

Outcome: median breast volume at 3 months [cc] post surgically, generalized linear model with negative binomial distribution		
Names	IRR^1^ (95%-CI^1^)	p-value
**Age:**	1.003 (1.000 to 1.007)	0.079
BMI* ≥ **30kg/m^2^**	0.898 (0.762 to 1.056)	0.200
**Nicotine consumption:**	1.013 (0.841 to 1.299)	0.849
**Complications, grade I:**	1.115 (0.891 to 1.376)	0.321
Average of MCN** distance at 12 months	0.999 (0.982 to 1.019)	0.951
Average of lower areola border to IMF*** distance at 12 months	1.055 (1.009 to 1.102)	**0.017**
Average of DA**** distance at 12 months	1.494 (1.095 to 2.024)	**0.009**
Average of DA**** distance at 12 months * complications, grade I	1.148 (1.038 to 1.1280)	**0.013**

Clear significant association between median breast volume, average distance of lower end of areola to IMF at 12 months (p = 0.017), and average areolar diameter (p = 0.009) respectively. GLM regression model with negative binomial distribution and a log link due to a skewed distribution. Significant interactions btw. the average of DA distance at 12 months and complications, grade I (p = 0.013). * Body Mass Index (BMI), MCN** = midclavicular distance, IMF*** = inframammary fold, DA**** = areolar diameter. ^1^ IRR = Incidence Rate Ratio, CI = Confidence Interval.

The results demonstrated a significant positive correlation between the median breast volume at 3 months and the average of lower areola border to IMF (p = 0.017, IRR: 1.055), and DA distance at 12 months post surgically (p = 0.009, IRR: 1.494), respectively. Furthermore, we identified significant interactions between the average of lower end of areola to IMF distance at 12 months and grade I complications (p = 0.013, IRR: 1.148). These results suggest that volumetric changes observed at 3 months postoperatively can predict subsequent lower pole expansion after reduction mammoplasty, indicating that higher postoperative breast volume was associated with greater subsequent inferior pole elongation.

### Association **btw. breast resection weight and changes in the lower breast pole at 12 months post-surgery**

We also examined potential correlations between breast resection weights and various predictive factors, including age, BMI ≥ 30 kg/m², nicotine use, and grade I complications, as well as associations with postoperative morphometric changes, such as the average MCN, the distance from the lower areola border to the IMF, and the DA distance at 12 months (Table 5).

**Table 5:** Associative regression results: average resection weight ranging from 500 to 2000 g [counts], with different risk factors.

Outcome: average resection weight ranging from 500 to 2000 g [counts], logistic regression model		
Names	Odds ratio (95%-CI^1^)	p-value
**Age:**	0.958 (0.927 to 0.990)	**0.010**
BMI* ≥ **30kg/m^2^**	7.606 (1.723 to 33.580)	**0.007**
**Nicotine consumption:**	1.521 (0.120 to 19.285)	0.746
**Complications, grade I:**	3.456 (0.841 to 14.210)	0.086
Average of MCN** distance at 12 months	0.777 (0.565 to 1.070)	0.123
Average of lower areola border to IMF*** distance at 12 months	0.522 (0.338 to 0.806)	**0.003**
Average of DA**** distance at 12 months	0.871 (0.296 to 2.558)	0.801

Positive correlations were found between larger resection weights ranging from 500 to 2000 g and average distances of lower areola border to IMF (p = 0.003), age (p = 0.010), and a BMI ≥ 30kg/m^2^ (p = 0.007). Logistic regression model for binary data. Estimates represent the log odds of " average resection weight ranging from 500 to 2000 g = yes" vs. " average resection weight ranging from 500 to 2000 g = no". * Body Mass Index (BMI). ^1^ CI = Confidence Interval.

The results showed a significant positive correlation between larger average resection weights (ranging from 500 to 2000 g) and the distance from the lower areola border to the IMF (p = 0.003, IRR: 0.522). Furthermore, significant associations were observed with age (p < 0.001, IRR: 1.391) and BMI ≥ 30 kg/m² (p < 0.001, IRR: 1.391).

These findings emphasize the importance of carefully selecting the breast resection weight during preoperative planning, as well as considering patient-specific factors such as age and obesity. Both factors can significantly impact postoperative morphometric changes and the aesthetic outcome of the breast following reduction mammoplasty.

## Discussion

This study shows that postoperative breast shape after superomedial-pedicle Wise-pattern reduction changes modestly but measurably across the first year. The predominant pattern was inferior-pole elongation with a subtle caudal migration of the NAC, whereas areolar diameter tended to stabilize or contract. Crucially, the early stabilized breast volume at 3 months independently correlated with subsequent lower-pole behavior. Patients with larger early postoperative volumes were more likely to exhibit greater inferior-pole elongation at later follow-up, consistent with a mild bottoming-out phenomenon. These findings are consistent with clinical experience that the lower pole is the principal driver of perceived ptosis or pseudoptosis after reduction mammaplasty and align with longitudinal morphometric literature. In line with previous work, volumetric assessment at 3 months has proven to be the most reliable indicator of early postoperative breast shape, as a discrete ptosis may develop thereafter, potentially diminishing the accuracy of later volumetric measurements.^2^^,^^3^^,^^16^ However, linear measurements obtained at 6 and 12 months could also offer valuable insight into progressive inferior-pole elongation and should therefore be used to assess bottoming-out over time.

Although the absolute morphometric changes observed were relatively modest, even small increases in inferior pole length may influence breast contour, lower pole fullness, nipple position, and patient perception of long-term aesthetic outcomes. Consequently, these measurements may have practical relevance for surgical planning and postoperative counseling despite their limited numerical magnitude.

By integrating standardized anthropometry with validated 3-dimensional (3D) surface imaging under posture-controlled acquisition, this work extends prior qualitative observations and provides a quantitative, clinically interpretable trajectory of shape change. 3D volumetry shows strong agreement with reference standards and excellent repeatability for volume and symmetry, supporting its use for planning and outcomes research.^2^^,^^9^^,^^16^ Two methodological pillars underpin our interpretation: (i) measurement posture materially affects volumetry – supine acquisitions systematically underestimate upright values – and (ii) seated/upright intraoperative volumetry with a handheld stereo-photogrammetric device is feasible and yields measurements commensurate with postoperative upright imaging.^14^^,^^17^ Within this framework, the 3-month visit coinciding with edema resolution and early soft-tissue accommodation functions as a pragmatic prognostic checkpoint for later contour behavior.

The non-linear volume trajectory observed across follow-up intervals likely reflects a combination of postoperative edema resolution, physiological weight fluctuations, and inherent variability of three-dimensional volumetric assessment. Therefore, volumetric measurements should be interpreted in conjunction with anthropometric measurements when evaluating long-term postoperative breast morphology.

The biomechanical plausibility of the observed associations is straightforward. A larger, heavier breast imposes a greater sustained tensile load on the lower-pole envelope; over time, viscoelastic creep and dermal remodeling incrementally lengthen the segment between the areola and the inframammary fold. Elevated BMI amplifies this demand and may coincide with attenuated dermal quality, while very large resections can exacerbate envelope–parenchyma mismatch.^25^ That the 3-month volume correlates with 12-month inferior-pole lengthening suggests that early stabilized size serves as a proxy for long-term mechanical demand on the redraped envelope and internal support structures. These relationships provide a practical framework for counseling: greater residual volume at three months was associated with increased inferior-pole elongation during follow-up.

Clinically, these data support a pragmatic care pathway anchored in posture-controlled 3D volumetry plus simple anthropometry at predefined intervals, with the 3-month visit used explicitly for risk stratification and expectation setting. When residual volume is comparatively high at three months, surgeons can anticipate greater lower-pole relaxation by one year, consider tighter lower-pole tailoring in similar future cases, and discuss staged approaches in higher-risk phenotypes. Intraoperatively, laser-level NAC alignment reduces vertical asymmetry and improves translation of planned targets into realized outcomes,^18^ while adopting upright/seated intraoperative volumetry minimizes posture-induced bias and improves the fidelity of early documentation.^14^^,^^17^ Together, these steps tie measurement rigor directly to operative decision-making and patient counseling about the trajectory, not just the endpoint, of postoperative change.

This analysis also helps reconcile apparently conflicting reports in the literature. Comparative series suggest superomedial designs often preserve upper-pole projection and NAC position more favorably than alternatives.^10^^,^^20^ However, heterogeneity in definitions, posture, and follow-up timing has limited direct synthesis. By focusing on a single, widely used technique and enforcing standardized, posture-controlled measurements, the present study quantifies how much drift to expect and which patients are most likely to experience it, moving beyond binary success/failure labels toward magnitude-and-risk estimates that map to everyday surgical choices.

Subgroup and sensitivity considerations further support robustness. The association between early volume and later inferior-pole behavior persisted after adjustment for patient and procedure covariates, and directional trends were consistent across BMI strata and resection sizes – patterns that cohere with a load-dependent mechanism. Although areolar diameter tended to contract overall, the magnitude was small and clinically neutral for most patients, suggesting that lower-pole length and NAC height are the more sensitive levers for perceived contour “aging.” This prioritization aligns with classic breast measurement literature and with contemporary 3D validation studies that emphasize volume and vertical landmark control as the strongest correlates of aesthetic symmetry.^2^^,^^9^^,^^15^^,^^16^^,^^18^^,^^19^^,^^21^

We acknowledge several limitations. The single-center design and technique uniformity are strengths for internal validity but may limit the generalizability of our findings to other pedicle techniques, skin resection patterns, or postoperative care protocols. As this study was retrospective and exploratory, no a priori sample size calculation was performed, and the study may therefore have been underpowered to detect smaller associations. Nevertheless, this cohort represents one of the larger longitudinal three-dimensional morphometric analyses of superomedial pedicle Wise-pattern reduction mammoplasty currently available. Postoperative BMI and body weight were not routinely documented during follow-up and therefore could not be incorporated into the regression analyses. Consequently, postoperative weight changes may have influenced breast morphology and represent a potential source of residual confounding. Additional unmeasured factors, including tissue quality, parenchymal suspension techniques, bra adherence, and dermal characteristics, may also have influenced the observed morphometric changes despite multivariable adjustment. Even with posture-controlled imaging, supine and upright volumetric measurements are not interchangeable, underscoring the importance of standardized patient positioning when comparing breast volumes over time.^17^ Finally, although objective morphometric measurements were obtained at predefined follow-up intervals, the inclusion of validated patient-reported outcome measures would further improve the clinical interpretation of the observed morphometric changes.

Looking forward, multi-center prospective studies should externally validate the 3-month volumetry checkpoint as an early surrogate for later morphology, using harmonized upright imaging posture and scheduled assessments (e.g., 6–8 weeks, 3/6/12/24 months). Predictive tools that combine early volume with covariates (BMI, resection weight, minor wound events) warrant development with rigorous calibration, decision-curve analysis, and independent validation. Interventional trials in higher-risk phenotypes can test technique-level modifiers of lower-pole support (internal suspension, selective envelope tailoring) and bundles of intraoperative standardization (including laser-level NAC alignment), while coupling morphometric trajectories to patient-reported outcomes to define clinically meaningful change. Advances in automated 3D workflows (landmark detection, segmentation) could reduce operator dependence and enable scalable, posture-consistent follow-up in routine practice.

## Conclusion

Postoperative breast shape following superomedial-pedicle Wise-pattern reduction evolves gradually over time. A **larger early postoperative volume at 3 months** appears to be the strongest practical predictor of subsequent lower-pole lengthening (“bottoming-out”), with BMI and resection weight providing additional risk context. Routine **upright** 3D volumetry combined with standardized anthropometric measurements and intraoperative laser level projection for symmetrical NAC-positioning offers a reproducible approach to forecast postoperative trajectories, refine surgical technique, and establish realistic expectations in everyday clinical practice.

## Financial disclosure statement

The authors have no financial interest in any of the products, devices, or drugs mentioned in this manuscript.

## STROBE-Statement

In accordance with the Journal of Plastic, Reconstructive & Aesthetic Surgery (JPRAS) commitment to standardized reporting, we confirm that this study adheres to the STROBE guidelines for cohort studies and patient series. All relevant aspects of the STROBE checklist have been addressed and implemented in the manuscript to ensure transparency and comprehensive reporting.

## Ethical approval

Not required.

## Funding

None.

## CRediT authorship contribution statement

**Thomas Holzbach:** Supervision, Methodology, Writing – review & editing. **Silke Graul:** Investigation, Data curation, Writing – review & editing. **Aljosa Macek:** Investigation, Data curation, Writing – review & editing. **Alvija Kucinskaite:** Investigation, Data curation, Writing – review & editing. **Alexander Kobler:** Investigation, Data curation, Writing – review & editing. **Sebastian Leitsch:** Methodology, Investigation, Writing – review & editing. **Rafael Loucas:** Conceptualization, Methodology, Supervision, Investigation, Writing – review & editing. **Michael-Alexander Pais:** Conceptualization, Methodology, Formal analysis, Investigation, Data curation, Writing – original draft, Writing – review & editing.

## Conflicts of interest

None declared.

## Appendix Supplementary materials

Image, application 1
