# Supplementary material for: Morphometric changes after superomedial pedicle wise pattern reduction mammoplasty – what we plan and where we end up
Source: JPRAS Open. 2026 Aug 25;52:300–9. doi: 10.1016/j.jpra.2026.08.031 (PMC13578466; doi:10.1016/j.jpra.2026.08.031)
Supplement: Supplementary file 1 [file mmc1.docx]

**STROBE Statement – Checklist of items that should be included in reports of cohort studies**

**Manuscript Title:**
Morphometric changes after superomedial pedicle wise pattern reduction mammoplasty – what we plan and where we end up

**Journal:** Journal of Plastic, Reconstructive & Aesthetic Surgery (JPRAS)

**TITLE AND ABSTRACT**

**Item 1a – Study design in title/abstract**
The study design (retrospective cohort study) is described in the abstract.

**Item 1b – Informative abstract**
The abstract provides a structured summary of background, methods, results, and conclusions, including statistical methods and main findings.

**INTRODUCTION**

**Item 2 – Background/rationale**
Scientific background and rationale are described in the Introduction, including current knowledge gaps in morphometric evolution after reduction mammoplasty.

**Item 3 – Objectives**
Specific objectives are stated at the end of the Introduction, including assessment of morphometric changes and analysis of associations with clinical variables.

**METHODS**

**Item 4 – Study design**
Retrospective cohort design stated in Methods (Section 2).

**Item 5 – Setting**
Single-center study conducted at Thurgau Hospital Group, Switzerland; inclusion period beginning July 2023; predefined follow-up at 3, 6, and 12 months.

**Item 6 – Participants**
Eligibility criteria: female patients ≥17 years undergoing primary bilateral superomedial pedicle Wise-pattern reduction mammoplasty.
Exclusion criteria: incomplete data, prior breast surgery.
Consecutive inclusion described.

**Item 7 – Variables**
Clearly defined variables:

- Anthropometric measures
- Postoperative volumetry
- Resection weight categories
- BMI ≥30 kg/m²
- Comorbidities
- Complication grading (Clavien–Dindo)
- Morphometric changes over time

**Item 8 – Data sources/measurement**
Anthropometry via standardized measurement sheet.
3D volumetry via Vectra H2 device in upright posture using predefined imaging protocol.

**Item 9 – Bias**
Selection bias minimized by consecutive inclusion.
Measurement bias reduced via standardized documentation and posture-controlled imaging.
Residual confounding acknowledged.

**Item 10 – Study size**
Study size determined by number of consecutive eligible patients with complete follow-up during study period; no formal sample size calculation performed.

**Item 11 – Quantitative variables**
Continuous and categorical variables defined; resection weight categorized (100–499 g vs. 500–2000 g); BMI categorized ≥30 kg/m².

**Item 12a – Statistical methods**
Descriptive statistics via frequency distributions.
Associations analyzed using generalized linear models (negative binomial distribution with log link) and logistic regression.
IRRs calculated.

**Item 12b – Subgroups/interactions**
Interactions analyzed between 3-month volume and morphometric changes; associations with age, BMI, resection weight, and complications.

**Item 12c – Missing data**
Only complete datasets included; no imputation performed.

**Item 12d – Loss to follow-up**
Only patients with complete predefined follow-up intervals included in final analysis.

**Item 12e – Sensitivity analyses**
Multivariable modeling performed to account for relevant covariates.

**RESULTS**

**Item 13a – Participant numbers**
100 consecutive eligible patients included in final analysis.

**Item 13b – Reasons for non-participation**
Patients excluded due to incomplete data or prior breast surgery.

**Item 14a – Descriptive data**
Baseline characteristics and comorbidities presented (Table 1).

**Item 14b – Missing data**
Analysis restricted to complete datasets.

**Item 14c – Follow-up time**
Predefined follow-up at 3, 6, and 12 months.

**Item 15 – Outcome data**
Reported postoperative volumetric data and morphometric changes at 3, 6, and 12 months.

**Item 16 – Main results**
IRRs and p-values reported for associations between postoperative volume, resection weight, BMI, age, and morphometric changes.

**Item 17 – Other analyses**
Multivariable analyses and interaction assessments described.

**DISCUSSION**

**Item 18 – Key results**
Summary of principal findings provided.

**Item 19 – Limitations**
Single-center design, retrospective nature, and potential residual confounding acknowledged.

**Item 20 – Interpretation**
Findings interpreted cautiously within context of biomechanical plausibility and existing literature.

**Item 21 – Generalisability**
External validity discussed, including applicability to other pedicle techniques.

**OTHER INFORMATION**

**Item 22 – Funding**
Funding: None declared.
Conflicts of interest: None declared.
